# DNA hypomethylation-related overexpression of SFN, GORASP2 and ZYG11A is a novel prognostic biomarker for early stage lung adenocarcinoma

**DOI:** 10.18632/oncotarget.26676

**Published:** 2019-02-26

**Authors:** Ryan Edbert Husni, Aya Shiba-Ishii, Tomoki Nakagawa, Tomoko Dai, Yunjung Kim, Jeongmin Hong, Shingo Sakashita, Noriaki Sakamoto, Yukio Sato, Masayuki Noguchi

**Affiliations:** ^1^ Doctoral Program in Biomedical Sciences, Graduate School of Comprehensive Human Sciences, University of Tsukuba, Ibaraki, Japan; ^2^ Department of Diagnostic Pathology, Faculty of Medicine, University of Tsukuba, Ibaraki, Japan; ^3^ Department of Thoracic Surgery, Faculty of Medicine, University of Tsukuba, Ibaraki, Japan

**Keywords:** epigenetics, DNA demethylation, early-stage lung adenocarcinoma, GORASP2, ZYG11A

## Abstract

Although alteration of DNA methylation in advanced cancer has been extensively investigated, few data for early-stage lung adenocarcinoma are available. Here, we compared DNA methylation profiles between adenocarcinoma *in situ* (AIS) and early invasive adenocarcinoma using the Infinium methylation array to investigate methylation abnormalities causing early progression of adenocarcinomas. We focused on differentially methylated sites which were located in promoter CpG islands or shore regions, and identified 579 hypermethylated sites and 23 hypomethylated sites in early invasive adenocarcinoma relative to AIS and normal lung. These hypermethylated genes were significantly associated with neuronal pathways such as the GABA receptor and serotonin signaling pathways. Among the hypomethylated genes, we found that GORASP2, ZYG11A, and SFN had significantly lower methylation rates at the shore regions and significantly higher protein expression in invasive adenocarcinoma. Moreover, overexpression of those proteins was strongly associated with patient’s poor outcome. Despite DNA demethylation at the promoter region might be rare relative to DNA hypermethylation, we identified 2 new genes, GORASP2 and ZYG11A, which show hypomethylation and overexpression in invasive adenocarcinoma, suggesting that they have important functions in tumor cells. These genes may be clinically applicable as prognostic indicators and could be potential novel target molecules for drug development.

## INTRODUCTION

Lung cancer is the leading cause of cancer death in men and the second-most common cause in women worldwide. Among the various histological subtypes, adenocarcinoma is the most frequent and its incidence is still increasing [1–3]. Lung adenocarcinogenesis shows multi-step progression starting from a pre-cancerous lesion, adenomatous atypical hyperplasia (AAH), and then to adenocarcinoma *in situ* (AIS), minimally invasive adenocarcinoma (MIA), and finally invasive lung adenocarcinoma, which has a poorer outcome [4].

Although many targeted drugs have already been established and are effective in patients with specific genetic aberrations, most tumors develop resistance to them, especially in the advanced stage, and therefore the mortality rate has not declined [5–7]. We have speculated that this is because such advanced tumors harbor numerous genetic abnormalities, i.e. they have a high mutation burden. On the other hand, in comparison with advanced lung adenocarcinoma, early-stage adenocarcinoma is thought to contain a relatively small number of genetic alterations, mostly of an epigenetic rather than a genetic type [8]. However, studies of early-stage lung adenocarcinoma have been scarce and knowledge is still limited. Therefore, discovery of epigenetic and genetic aberrations in early-stage lung adenocarcinomas such as AIS would help to clarify the molecular carcinogenesis of such tumors.

We have extensively studied the gene expression profiles of early stage adenocarcinoma and found several genes that are overexpressed in early invasive tumors but not in adenocarcinoma *in situ*. These genes include stratifin (SFN) [9], immunoglobulin binding protein 1 (IGBP1), [10] ovarian carcinoma immunoreactive antigen domain 2 (OCIAD2), [11] and dimethylarginine dimethylaminohydrolase 2 (DDAH2) [12]. In the course of our investigations, we realized that overexpression of all these genes does not occur through genetic alteration but is associated with epigenetic abnormality.

Abnormal DNA methylation is an important component of epigenetic change and has been reported to correlate with the progression of human carcinomas [13, 14]. Hypermethylation can silence the expression of tumor suppressor genes and result in tumor progression [15]. Recent study has also emphasized the importance of hypermethylation in lung cancer as a potential biomarker and drug target [16]. On the other hand, global hypomethylation of genomic DNA can lead to genetic alteration [17] as well as overexpression of proto-oncogenes and growth factors, [18] resulting in tumorigenesis. Our group has shown that overexpression of SFN is correlated with DNA hypomethylation status. Although the promoter region of SFN is totally methylated in normal lung and AIS, most invasive adenocarcinomas show partial methylation, suggesting that demethylation at this site leads to overexpression of SFN [19].

Based on these findings for SFN and the importance of epigenetic alteration in lung adenocarcinoma, we have been focusing on abnormalities of DNA methylation in early-stage lung adenocarcinomas with the expectation that some oncogenes would become demethylated and thus overexpressed, leading to tumor progression.

The purpose of the present study was to screen exhaustively for differentially methylated genes in early-stage lung adenocarcinomas, and identify those that are associated with lung adenocarcinogenesis and early progression.

## RESULTS

### Infinium methylation array, pathway analysis and candidate gene selection

One of the methods to find differentially methylated genes is using Infinium methylation array [20]. Methylation array using the Infinium MethylationEPIC Bead Chip microarray (Illumina) analyzed more than 850,000 CpG sites covering 99% of the Refseq genes [21]. For further processing we used the MACON web tool. We determined the methylation rate in terms of the β value [22].

**Figure 1:**
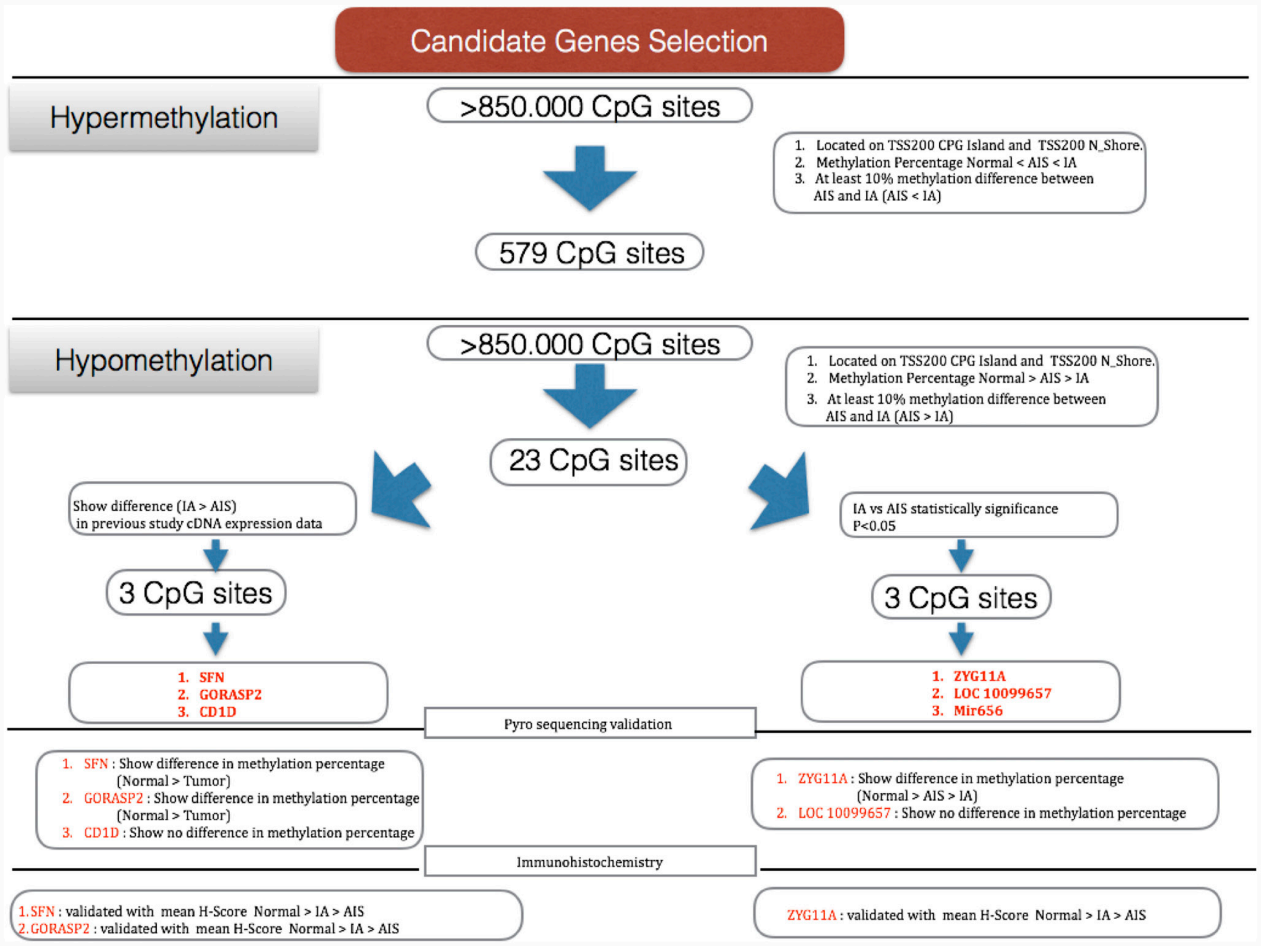
Selection of candidate genes AIS: adenocarcinoma *in situ*, IA: invasive adenocarcinoma. Several conditions were used to narrow down the candidates from >850,000 CpG sites: location of CpG sites at the transcription start site (TSS) 200 island and N_shore; a methylation difference of at least 10% between AIS and invasive adenocarcinoma; highest methylation percentage in normal tissue, followed by AIS and invasive adenocarcinoma for the hypomethylation group, and *vice versa* for the hypermethylation group. On the basis of these conditions we selected 23 CpG sites for the hypomethylation group and 579 for the hypermethylation group, which are differentially methylated regions (DMR) between the groups.

It has long been known that DNA methylation at CpG islands in the gene promoter region regulates the expression of downstream genes. Thus, to narrow down and select suitable gene candidates we adopted several conditions for hypomethylated gene screening: location of the CpG sites in promoter CpG island or shore regions between the transcription start site (TSS) and 2 kb upstream from the TSS; a methylation difference of more than 10% between AIS and invasive adenocarcinoma methylation rate being highest in normal lung and then decreasing stepwise to AIS, and then invasive adenocarcinoma. Opposite conditions were used for screening of hypermethylated genes. From the given conditions we selected 23 CpG sites as hypomethylated genes and 579 CpG sites as hypermethylated genes (519 sites in CpG island, 60 sites at shore region, Supplementary Table 1), which were in a differentially methylated region (DMR) between AIS and invasive adenocarcinoma (Figure 1). Using 579 CpG sites that showed hypermethylation in invasive adenocarcinoma, pathway analysis was performed to investigate significantly associated pathways (Supplementary Tables 2, 3). The results from both pathway analysis tools consistently indicated that hypermethylation frequently occurs in neural-related pathways such as GABA receptor signaling and serotonin receptor signaling.

Although the number of hypomethylated genes was relatively limited in comparison to that of hypermethylated genes, we expected that these genes might include important oncogenes showing overexpression induced by DNA demethylation, and focused on 23 CpG sites that showed hypomethylation (5 sites in CpG island, 18 sites at shore region, Supplementary Table 4).

For further selection, we performed statistical analysis between AIS vs. invasive adenocarcinoma and selected 3 CpG sites, ZYG-11 family member A (ZYG11A), LOC10099657, and Mir656 (*p* < 0.05) as genes that show a significantly lower methylation rate in invasive adenocarcinoma relative to AIS. However, because we planned to validate the results using IHC, we considered that microRNAs would not be appropriate candidates for investigation, and therefore excluded Mir656 on this basis. On the other hand, we previously conducted cDNA microarray analysis and obtained expression profiles of AIS and invasive adenocarcinoma (Supplementary Table 5). Here, we compared the methylation profiles with RNA expression profiles to identify genes whose RNA expression was higher in invasive adenocarcinoma than in AIS [23]. Consequently, 3 CpG sites, SFN, Golgi reassembly-stacking protein 2 (GORASP2), and cluster of differentiation 1 (CD1D) were selected (Table 1). Those 5 candidate sites were located at the shore regions (Figure 1).

**Table 1:** Six candidate genes revealed using the array

				Beta value							
									Mean	Mean	Meth
Gene name	Normal			AIS			Invasive		AIS	Invasive	Diff
	1	2	1	2	3	1	2	3			
ZYG11A	0.72	0.74	0.8	0.71	0.64	0.52	0.51	0.56	0.72	0.53	0.19
SFN	0.59	0.63	0.36	0.55	0.46	0.25	0.27	0.41	0.46	0.31	0.15
CD1D	0.81	0.84	0.84	0.81	0.76	0.86	0.71	0.52	0.8	0.70	0.11
LOC10099657	0.63	0.66	0.36	0.54	0.46	0.18	0.27	0.19	0.45	0.21	0.24
GORASP2	0.87	0.83	0.8	0.77	0.61	0.82	0.44	0.44	0.73	0.57	0.16
MIR656	0.77	0.8	0.76	0.74	0.75	0.71	0.62	0.63	0.75	0.65	0.1

Beta value represents the methylation percentage on a scale of 0-1. All 6 genes show at least a 10% methylation difference between AIS and invasive adenocarcinoma.

### Pyrosequencing

**Figure 2:**
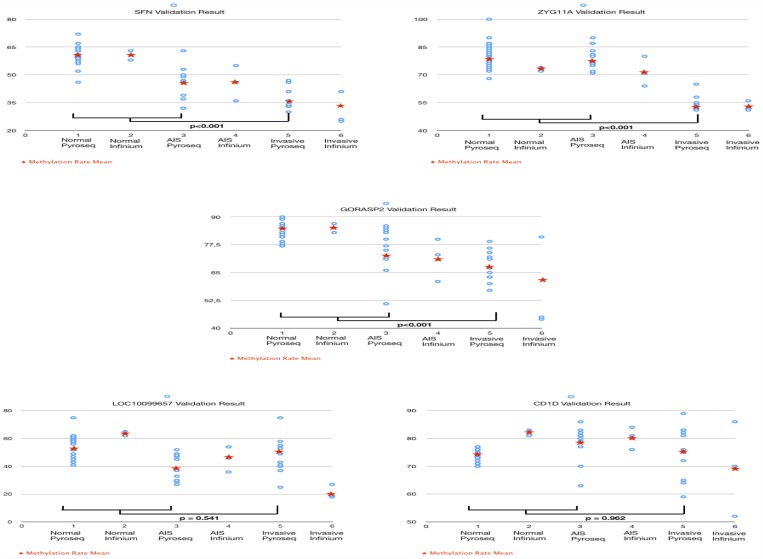
Pyrosequencing DNA methylation rates of SFN, GORASP2, ZYG11A, LOC10099657, and CD1D were validated using pyrosequencing for another 11 frozen samples of invasive adenocarcinoma, 10 samples of AIS, and 21 samples of normal lung tissue. While LOC10099657 (*p* = 0.541) and CD1D (*p* = 0.962) showed no significant difference of methylation rate among invasive adenocarcinoma, AIS, and normal lung, the other 3 genes showed a significantly lower methylation rate in invasive adenocarcinoma relative to AIS or normal lung (*p* < 0.001).

We employed pyrosequencing to validate the DNA methylation levels of genes we had selected based on the results of methylation array [24]. We calculated the mean methylation rate from all 5 candidate genes. While we found a significant difference in the methylation rate between AIS and invasive adenocarcinoma (Normal>AIS>Invasive) for SFN, GORASP2, and ZYG11A, no difference was evident for CD1D and LOC10099657 (Figure 2). In addition, we also performed pyrosequencing using the same 8 samples that we used for the methylation array. While the methylation rates were not exactly the same, both results showed similar tendency that invasive adenocarcinoma had lower methylation rate than AIS or normal lung (Supplementary Figure 1).

### Association of methylation rate with protein expression

We carried out IHC to validate and find correlations between methylation status and protein expression for ZYG11A, GORASP2, and SFN [25]. The H-score was calculated and then compared with the methylation rate obtained from pyrosequencing.

**Figure 3:**
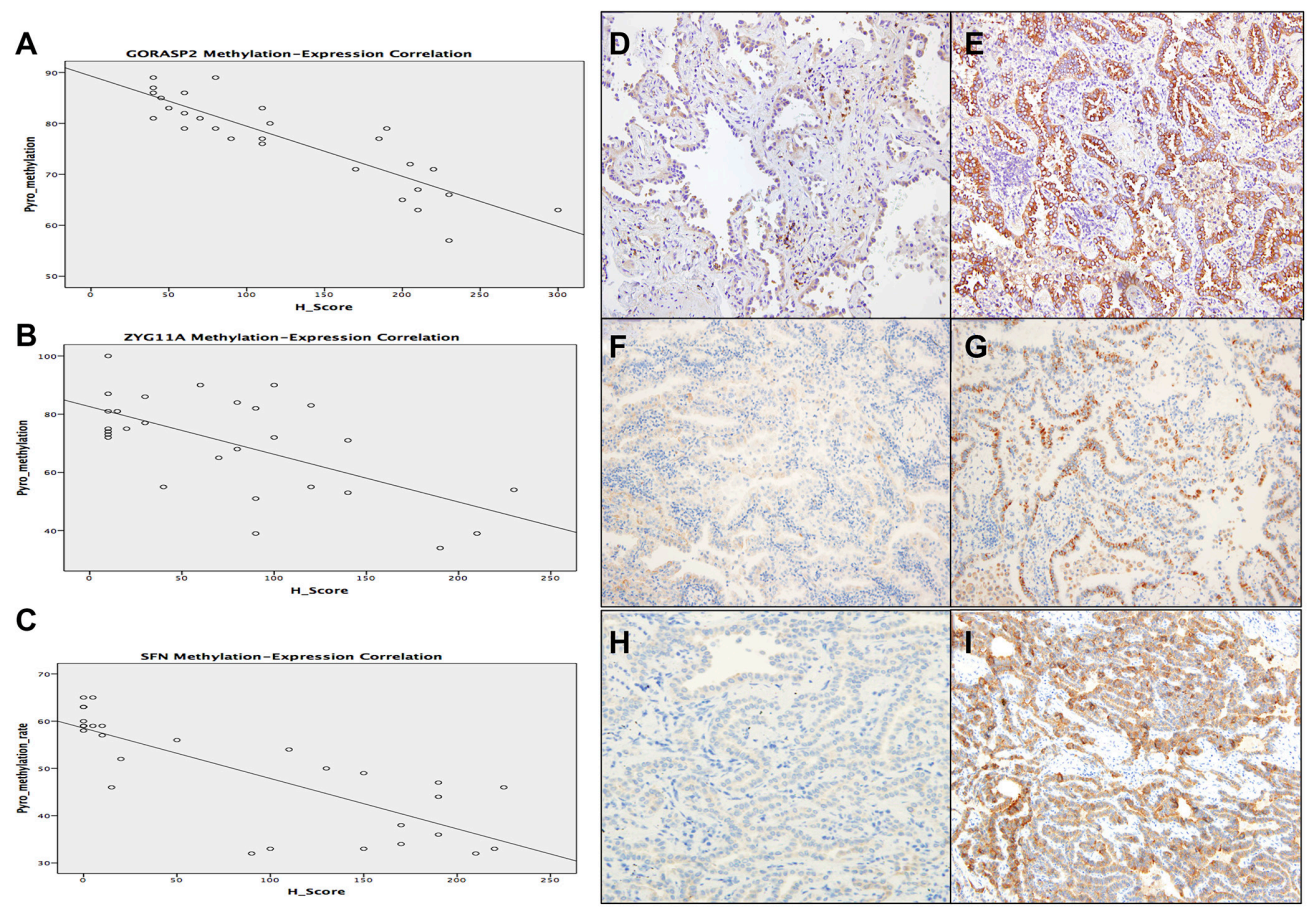
GORASP2, ZYG11A, and SFN methylation-expression correlation and IHC staining pattern GORASP2 (r: −0.878, *p* < 0.001), ZYG11A (r: −0.623, *p* < 0.001), and SFN (r: −0.793, *p* < 0.001) showed a statistically significant negative linear relationship between the H-score and the methylation rate (**A**–**C**). Representative cases with weak and strong expression of GORASP2 (**D**, **E**), ZYG11A (**F**, **G**), SFN (**H**, **I**).

All three genes showed a statistically significant negative linear relationship between the H-score and the methylation rate (Figure 3A–3C). The mean H-score also showed the lowest value in normal lung, followed in order by AIS and then invasive adenocarcinoma (61<162<208 for GORASP2; 33<94<131 for ZYG11A; 10<154<184 for SFN). These results suggest that DNA methylation status might regulate the protein expression of GORASP2, ZYG11A, and SFN.

### Expression pattern of GORASP2, ZYG11A and SFN in lung adenocarcinoma

Next, to investigate the expression pattern and clinicopathological implications of SFN, GORASP2 and ZYG11A, we performed IHC using 171 cases of lung adenocarcinoma (Figure 3D–3I).

SFN positivity was detected in 29/46 (63%) lepidic, 13/26 (52%) papillary, 10/19 (52%) acinar, and 27/32 (84%) solid adenocarcinomas. Among non-invasive lung adenocarcinomas, strong expression was detected in 5/19 (26%) AIS and 13/29 (45%) MIA (Supplementary Table 6). SFN expression showed significant correlations with lymphatic permeation, vascular invasion, and pathological subtype of lung adenocarcinoma (Supplementary Table 6).

Among invasive adenocarcinomas, strong expression of GORASP2 was detected in 18/46 (39%) lepidic, 22/26 (84%) papillary, 11/19 (57%) acinar, and 25/32 (78%) solid adenocarcinomas. Among non-invasive lung adenocarcinomas, strong expression was detected in 0/19 (0%) AIS and 2/29 (7%) MIA (Supplementary Table 7). Although GORASP2 expression showed no significant correlation with age, it was significantly correlated with pathological stage, lymphatic permeation, vascular invasion, and pathological subtype of lung adenocarcinoma (Supplementary Table 7). GORASP2 showed significantly higher expression in invasive lung adenocarcinoma (76/123 cases, 61%) than in non-invasive lung adenocarcinomas (2/48 cases, 4%).

On the other hand, strong expression of ZYG11A was detected in 17/46 (37%) lepidic, 16/26 (61%) papillary, 13/19 (68%) acinar, and 16/32 (50%) solid adenocarcinomas. Among non-invasive lung adenocarcinomas, strong expression was detected in 0/19 (0%) AIS and 11/29 (38%) MIA (Supplementary Table 8). Although ZYG11A expression showed no significant correlation with age and sex, it was significantly correlated with pathological stage, lymphatic permeation, vascular invasion, and pathological subtype of lung adenocarcinoma (Supplementary Table 8). ZYG11A showed significantly higher expression in invasive lung adenocarcinoma (62/123 cases, 50%) than in non-invasive lung adenocarcinoma (11/48 cases, 22%).

### Correlation of GORASP2 and ZYG11A expression with overall postoperative survival

**Figure 4:**
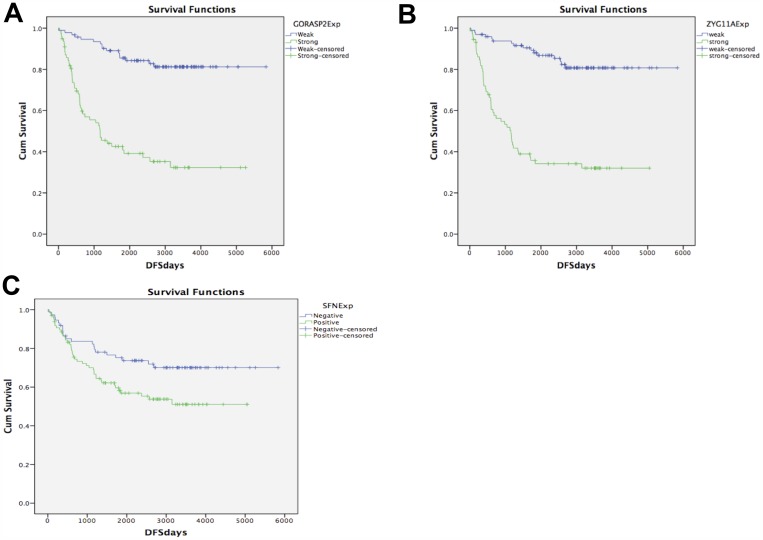
KM curve Disease-free survival depicted as Kaplan–Meier curves shows the correlation between GORASP2 (**A**), ZYG11A (**B**), and SFN (**C**) expression and outcome. Strong expression of GORASP2, ZYG11A, and SFN was associated with poor prognosis relative to weak expression (*p < 0.001).*

The Kaplan–Meier curves indicated that the patients in the strong GORASP2, ZYG11A or SFN expression group had a significantly poorer outcome than those in the weak expression group (Figure 4). Additionally, we divided the patients into stage I and II to find out the association between gene expression and prognosis in each stage [26]. Strong expression of GORASP2 or ZYG11A was significantly associated with poorer outcome in each stage (Supplementary Figure 2).

### Multivariate analysis using the Cox proportional hazards model

After adjustment for gender, age, pathological stage, vascular invasion, lymphatic permeation, GORASP2 expression, ZYG11A expression, and SFN expression, patients with strong expression of GORASP2 and ZYG11A showed a significantly higher risk of lung cancer-related death than those with weak expression {(HR: 0.332, 95% CI: 0.179–0.613, *P*: < 0.001) and (HR: 0.286, 95% CI: 0.155–0.527, *P*: < 0.001)}. Multivariate analysis also indicated that lymphatic permeation, vascular invasion, pathological stage, and expression of GORASP2 or ZYG11A were independent prognostic factors indicative of poor survival in patients with lung adenocarcinoma (Table 2).

**Table 2:** Multivariate analysis

Clin. features	Univariate analysis			Multivariate analysis		
	HR	95% CI	*P* value	HR	95% CI	*P* value
Gender	0.394	0.220–0.704	0.002	0.791	0.425–1.473	0.460
Age	1.091	0.625–1.903	0.760			
Vascular Invasion	0.13	0.072–0.234	<0.001	0.272	0.133–0.555	<0.001^*^
(- vs +)						
Lymphatic Permeation	0.194	0.115–0.326	<0.001	0.420	0.236–0.747	0.003^*^
(− vs +)						
GORASP2 Exp	0.173	0.098–0.306	<0.001	0.332	0.179–0.613	<0.001^*^
(Weak vs Strong)						
ZYG11A Exp	0.161	0.091–0.285	<0.001	0.286	0.155–0.527	<0.001^*^
(Weak vs Strong)						
SFN Exp	0.555	0.328–0.938	0.028	1.427	0.819–2.487	0.209
(− vs +)						
Pathological Stage	0.219	0.132–0.364	<0.001	0.534	0.316–0.903	0.019^*^
(1 vs other)						

Adjusted for gender, age, GORASP2 expression, ZYG11A expression, SFN expression, vascular invasion, lymphatic permeation, and pathological stage.

## DISCUSSION

DNA methylation is one of the most important epigenetic alterations related to tumor initiation and progression. There are two forms of DNA methylation phenomena: hypermethylation and hypomethylation [14, 27] Many tumor tissues show global hypomethylation and regional hypermethylation, particularly in tumor suppressor genes. However, according to our pathway analysis results, most hypermethylated genes were involved in neural pathways, and not tumor suppressor pathways. This finding indicated that the rate of methylation of these neural-related genes increased during tumor progression from the early to the advanced stage. Since it is generally known that expression of differentiation-related genes including neural markers is suppressed in cancer cells, we consider that hypermethylation in such pathways might be a natural phenomenon induced during the course of carcinogenesis. Notably, the result of KEGG pathway analysis indicated that hypermethylated genes were enriched in “transcriptional misregulation in cancer” (MEF2C, WNT16, BAIAP3, TFE3, TSPAN7, GRIA3, HIST1H3G, WT1, TLX1, MYCN) (Supplementary Figure 3) suggesting that this may be one of the direct causes of carcinogenesis.

On the other hand, since we had previously demonstrated that the SFN promoter region was hypomethylated in invasive adenocarcinoma, leading to its overexpression, this fact motivated us to seek other genes also showing promoter hypomethylation. If overexpressed genes can be found in tumors, they might serve as novel prognostic indicators or potential targets for drug development. Consequently, although we selected 579 CpG sites that showed hypermethylation in invasive adenocarcinoma relative to AIS and normal lung, only 23 of them showed hypomethylation in invasive adenocarcinoma, suggesting that DNA demethylation around the promoter region might be rare relative to DNA hypermethylation. Despite this, we identified 2 new genes, GORASP2 and ZYG11A, which show hypomethylation and overexpression in invasive adenocarcinoma, suggesting that they have important functions in tumor cells. In our previous studies, we found that IGBP1, OCIAD2 and DDAH2 showed high expression in lung adenocarcinoma and their expression appeared to be epigenetically regulated. However, those genes were not included in the candidate list here. As we reported, overexpression of IGBP1 is due to dysregulation of particular microRNAs [10]. Although the mechanism of OCIAD2 and DDAH2 overexpression is still unclear, we expect another epigenetic events such as histon modification or non-coding RNA might be associated. Additionally, recent study revealed that DAPK2, MFSD2A, THSD1 and WNT7A were oncogenes which showed high expression and low methylation in lung adenocarcinoma [28]. While we did not select these genes as candidates, this may be because the samples we used here were early stage tumors, which has a different genetic or epigenetic features from advanced tumors.

We found that expression of GORASP2, ZYG11A, and SFN had a statistically significant negative linear relationship with methylation status, and that invasive adenocarcinoma showed significantly higher expression of both genes than AIS. Also, we found that strong expression of both genes was associated with poor prognosis. Finally, we revealed that both genes are independent prognostic factors for lung adenocarcinoma.

The Golgi apparatus is an organelle whose main function is glycosylation, packaging and transport of synthesized proteins from the endoplasmic reticulum. The secretory vesicles are then distributed to the plasma membrane, lysosomes, or extracellular space [29]. The GORASP2 gene encodes the membrane protein Golgi reassembly-stacking protein of 55 kDa (GRASP55), also known as GORASP2 protein, whose function is to stack and link the Golgi cisternae, thus playing an important role in the formation and membrane dynamics of the Golgi apparatus [30–32]. A recent study has confirmed the importance of the Golgi apparatus in overcoming the acquired resistance of lung adenocarcinoma to EGFR tyrosine kinase inhibitors. Disrupting the Golgi apparatus via inhibition of ADP ribosylation factor 1 (ARF-1) exerts anti-tumor activity against EGFR-activated tumor cells [33]. Similarly, inhibition of GORASP2 might disrupt the membrane dynamics of the Golgi apparatus, thus leading to a substantial anti-tumor effect.

Studies of the ZYG11 gene in humans have been very limited, but in *Caenorhabditis elegans* the human homologue ZYG11 gene is important for cell division and embryonic development [34]. A further study has shown that the ZYG11 homologue is expressed in human sperm cells and functions in cell division during meiosis [35]. In humans, the ZYG11 gene family has three homologues, ZYG11A, ZYG11B, and ZER-1 [36]. One recent study has demonstrated an oncogenic function of the overexpressed ZYG11A gene in non-small cell lung cancer (NSCLC), being correlated with poor prognosis, in accordance with our data. It was also shown that depletion of ZYG11A suppressed cyclin E1 (CCNE1), thus suppressing cell cycle progression [37].

Recently we have reported that SFN stabilizes receptor tyrosine kinases such as EGFR and MET by facilitating their deubiquitination in lung adenocarcinoma cells. Since DNA demethylation-triggered overexpression of SFN has essential functions during the course of lung adenocarcinoma progression, we expect that GORASP2 and ZYG11A might also have some functional association in this tumor. We are planning to perform *in vitro* analysis to elucidate their functions, and we already confirmed their protein expression in several lung adenocarcinoma cell lines using Western blotting (data not shown).

In conclusion, we have found 579 hypermethylated sites and 23 hypomethylated sites in invasive adenocarcinoma. Hypermethylated genes are significantly associated with neural pathways such as the GABA receptor and serotonin signaling pathways. Among the hypomethylated genes, we revealed that GORASP2, ZYG11A, and SFN had significantly lower methylation rates in invasive adenocarcinoma, leading to overexpression of their proteins. In particular, GORASP2 and ZYG11A appear to be novel independent prognostic factors in early invasive lung adenocarcinoma. Although further analysis is required to understand and clarify the function of both genes in lung adenocarcinogenesis, these genes might be clinically applicable as prognostic indicators and with potential application as target molecules in lung adenocarcinoma.

## MATERIALS AND METHODS

### Sample selection

For the Infinium methylation array, samples from 8 patients were collected at the University of Tsukuba Hospital (Ibaraki, Japan) in 2017. Three of the samples were AIS, another three were early but invasive adenocarcinoma, and the rest were normal lung samples collected from surgically resected pulmonary bullae. All six lung adenocarcinoma samples were collected by scratching directly from the freshly cut surface of the resected tumor. We confirmed cytologically that tumor cells formed the major component of the samples used. Genomic DNA was extracted from all the samples using a DNeasy Blood and Tissue kit (QIAGEN, Hilden, Germany).

For pyrosequencing, 42 samples were selected from specimens that had been surgically resected at the University of Tsukuba Hospital between 2015 and 2017; 21 samples of normal lung, 10 cases of AIS and 11 cases of invasive adenocarcinoma. We collected the tumor cells from cryosections (10 μm thick) using a laser microdissection system, LMD-6000 (Leica, Wetzlar, Germany).

For immunohistochemistry (IHC), we selected 14 formalin-fixed and paraffin-embedded (FFPE) samples which were also used for pyrosequencing. For clinicopathological analysis, we also added another 171 samples of lung adenocarcinoma surgically resected at the University of Tsukuba Hospital between 1999 and 2007, from which tissue microarray (TMA) blocks were constructed.

Informed consent for this study had been obtained from all of the patients.

### Infinium methylation array

Infinium methylation array analyses were performed using the Infinium MethylationEPIC Bead Chip microarray (Illumina, San Diego, CA, USA). Genomic DNA (1 μg) was treated with sodium bisulfite using a Zymo EZ DNA methylation kit (Zymo Research, Irvine, CA, USA) and the bisulfite-modified DNA was amplified prior to hybridization array. The array was scanned with an iScan System (Illumina).

To process the raw data obtained from the Infinium methylation array, we used the Methylation Array Computing Navigation (MACON) web tool. MACON performs probe filtering, beta mixture quantile (BMIQ) normalization, genomic block analysis and annotation, finally producing a data subset for further analysis [38].

### Pathway network analysis

Two different tools were used to analyze candidate genes from the methylation array to obtain associated cellular pathways for comparison. The first one is the Ingenuity Pathway Analysis (IPA) system, which automatically generates networks of gene, protein, small-molecule, and disease associations on the basis of data held in a proprietary database. Data were uploaded as an Excel file into the Ingenuity software (Ingenuity Systems, Redwood City, CA, USA) [39]. The second one is the Database for Annotation, Visualization and Integrated Discovery (DAVID) web tool, which is a high-throughput and integrated data-mining environment for analyzing the gene lists acquired from high-throughput genomic experiments [40]. Data were uploaded as a text file into the DAVID web tool. The pathway map was obtained by KEGG [41].

### Pyrosequencing

Bisulfite conversions were done prior to pyrosequencing by using the Epitect Bisulfite Kit (QIAGEN).

Primers used for PCR and pyrosequencing were designed using QIAGEN software, PyroMark Assay Design 2.0 (QIAGEN). The sequencing process used CpG assay and was performed using PyroMarkQ24 (QIAGEN) with suitable reagents from QIAGEN.

Pyrosequencing primers:

SFN-Sequence primer: AGGTGTTAGTGTAGG; SFN-Forward primer: TAGTTTGGAGTTTGGAAAGGTGTTAGTGTA; SFN-Reverse primer: CTACCTCCCTCTCCTCTCT-Biotin; GORASP2-Sequence primer: TTTGGGTTAGAATATTTTATGG; GORASP2-Forward primer: GAGGTAAGTTAATTATTTTGGGTTAGAA; GORASP2-Reverse primer: TCCCTCTAAAACCCCTAATTACT-Biotin; ZYG11A-Sequence primer: TGGTAGATATTTGTTAATATTTTGT; ZYG11A-Forward primer: GGGTTTTTTTTTTGGAGTAGGTTAT; ZYG11A-Reverse primer: ATTTCAAAAACTTTACCCCTAAAAATC-Biotin; LOC10099657-Sequence primer: GGTGTGGAGTTAATATTTATTT; LOC10099657-Forward primer: AGTGGTTAGGTGTGGAGTTAATAT; LOC10099657-Reverse primer: AAACACAACACCAACCATTTTATCA-Biotin; CD1D-Sequence primer: AGGTAGATATTAGGGTTAGA; CD1D-Forward primer: GGGGTGTGAGGTGATGTT; CD1D-Reverse primer: CCCTCTCCCTCACTCTTTTTATC-Biotin.

### Immunohistochemistry (IHC)

For IHC, we used 4-μm-thick sections cut from FFPE blocks and FFPE TMA blocks. The sections were deparaffinized and rehydrated. For antigen retrieval, we autoclaved the sections in 10 mmol/L citrate buffer (pH 6.0) at 105° C for 15 min. Further steps were performed on an automated stainer, Histostainer 48a (Nichirei Biosciences, Tokyo, Japan). Endogenous peroxidase was blocked with 3% hydrogen peroxide. The slides were incubated in a 1:100 dilution of rabbit polyclonal GORASP2 antibody (Atlas Antibodies, Bromma, Sweden), in a 1:50 dilution of rabbit polyclonal ZYG11A antibody (Atlas Antibodies), and in a 1:800 dilution of mouse monoclonal SFN antibody (Sigma-Aldrich, St. Louis, MO, USA) at RT for 1 hour, and subsequently incubated in the secondary antibody at RT for 1 hour. The signal was detected using DAB (Dako REAL Envision Detection System; Dako, Glostrup, Denmark). Hematoxylin was used for counterstaining. We evaluated all cases without prior knowledge of the clinicopathological data.

Evaluation of GORASP2, ZYG11A, and SFN staining was based on cytoplasmic staining, and the results were evaluated by the H-score system [42]. For survival analysis, the ROC curve was drawn to determine the best cut-off point of the score. Since the aim of the study was to clarify the correlation between GORASP2 and ZYG11A expression in relation to prognosis, we used GORASP2 and ZYG11A expression and patient outcome as variables for drawing the ROC curve. We drew a diagonal line from the bottom right to the top left corner of the curve, and the cut-off point was the coordinate where the diagonal line crossed over the curve. Here, for GORASP2, the coordinate was 185 (0.746 sensitivity and 0.287 (1-specificity)), and for ZYG11A it was 135 (0.762 sensitivity and 0.241 (1-specificity)), and this was adopted as the cut-off point in this study. Counts below the cut-off point were judged to represent weak expression, and those above as strong expression.

### Statistical analysis

For all statistical analyses, SPSS 22 (SPSS, Chicago, IL) and CLC Work Bench (Filgen, Aichi, Japan) were used. For determining the cut-off point for IHC scoring, the ROC curve method was used. Correlation of clinicopathological features with GORASP2 and ZYG11A expression was analyzed by chi-squared test. The Kaplan-Meier method was used for calculation of survival curves, and log-rank test was performed for comparisons. Multivariate analysis was done using the Cox proportional hazards model. Differences were considered statistically significant at *p* ≤ 0.05.

## SUPPLEMENTARY MATERIALS FIGURES AND TABLES





## Abbreviations

IHC: immunohistochemistry
AAH: adenomatous atypical hyperplasia
AIS: adenocarcinoma *in situ*
MIA: minimally invasive adenocarcinoma
GORASP2: Golgi reassembly-stacking protein 2
ZYG11A: ZYG-11 family member A
SFN: stratifin
