# Supplementary material for: DNA hypomethylation-related overexpression of SFN, GORASP2 and ZYG11A is a novel prognostic biomarker for early stage lung adenocarcinoma
Source: Oncotarget. 2019 Feb 26;10(17):1625–36. doi: 10.18632/oncotarget.26676 (PMC6422190; doi:10.18632/oncotarget.26676)
Supplement: Supplementary file 1 [file oncotarget-10-1625-s001.pdf]

DNA hypomethylation-related overexpression of SFN, GORASP2 and ZYG11A is a novel prognostic biomarker for early stage lung adenocarcinoma

SUPPLEMENTARY MATERIALS

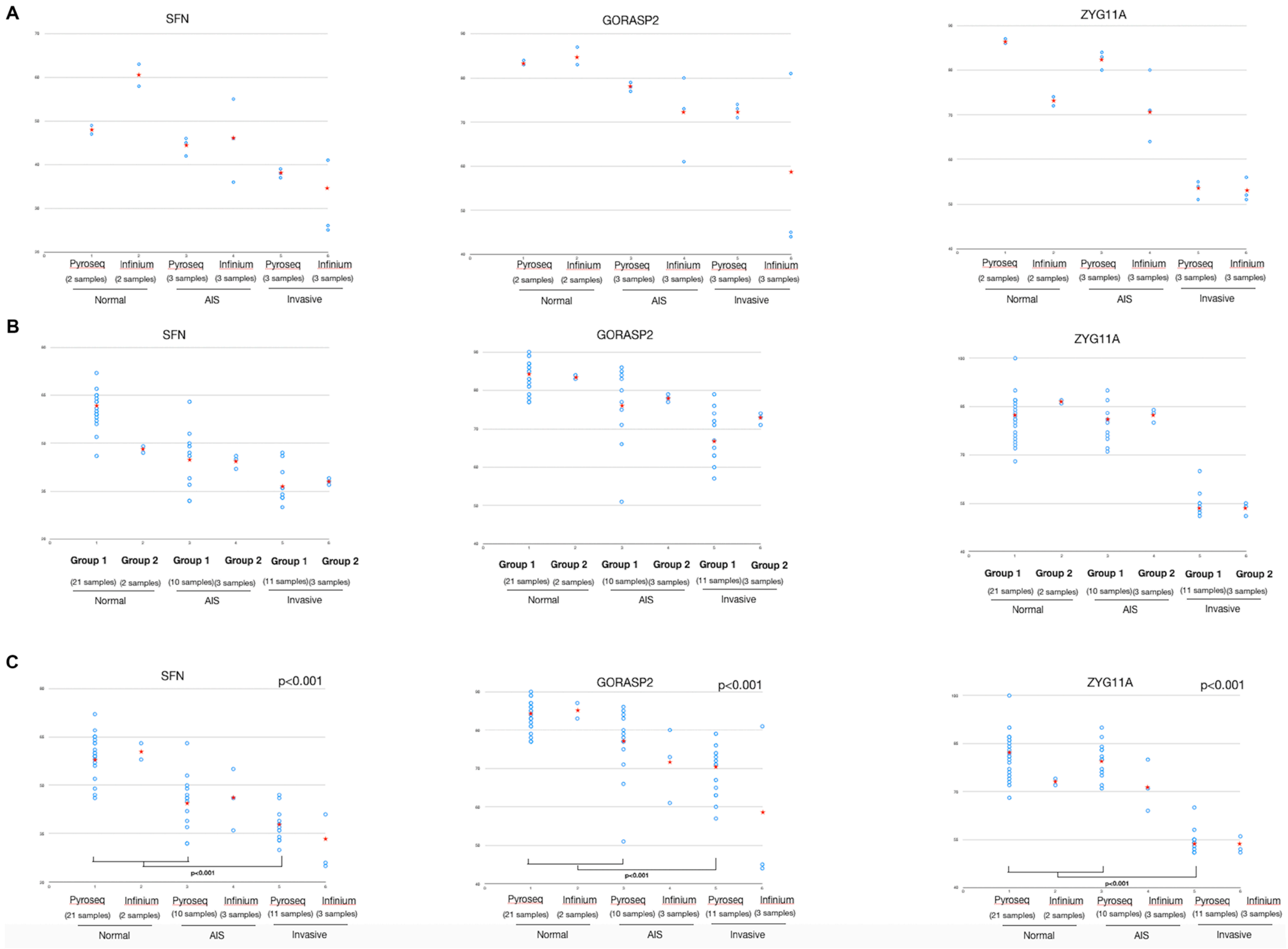

Supplementary Figure 1: Methodological comparison between infinium methylation array and pyrosequencing. (**A**) 8 samples were subjected to Infinium methylation array and pyrosequencing. Each graph shows the comparison of the results in SFN, GORASP2, and ZYG11A between array analysis and pyrosequencing. (**B**) Additionally to the 8 samples, we performed pyrosequencing using new 42 samples. Each graph compares the results between group 1 (8 samples that we used for Infinium) and group 2 (new 42 samples). (**C**) We combined the results of 8 and new 42 samples as a final result of pyrosequencing, and compared with the results of Infinium methylation array.

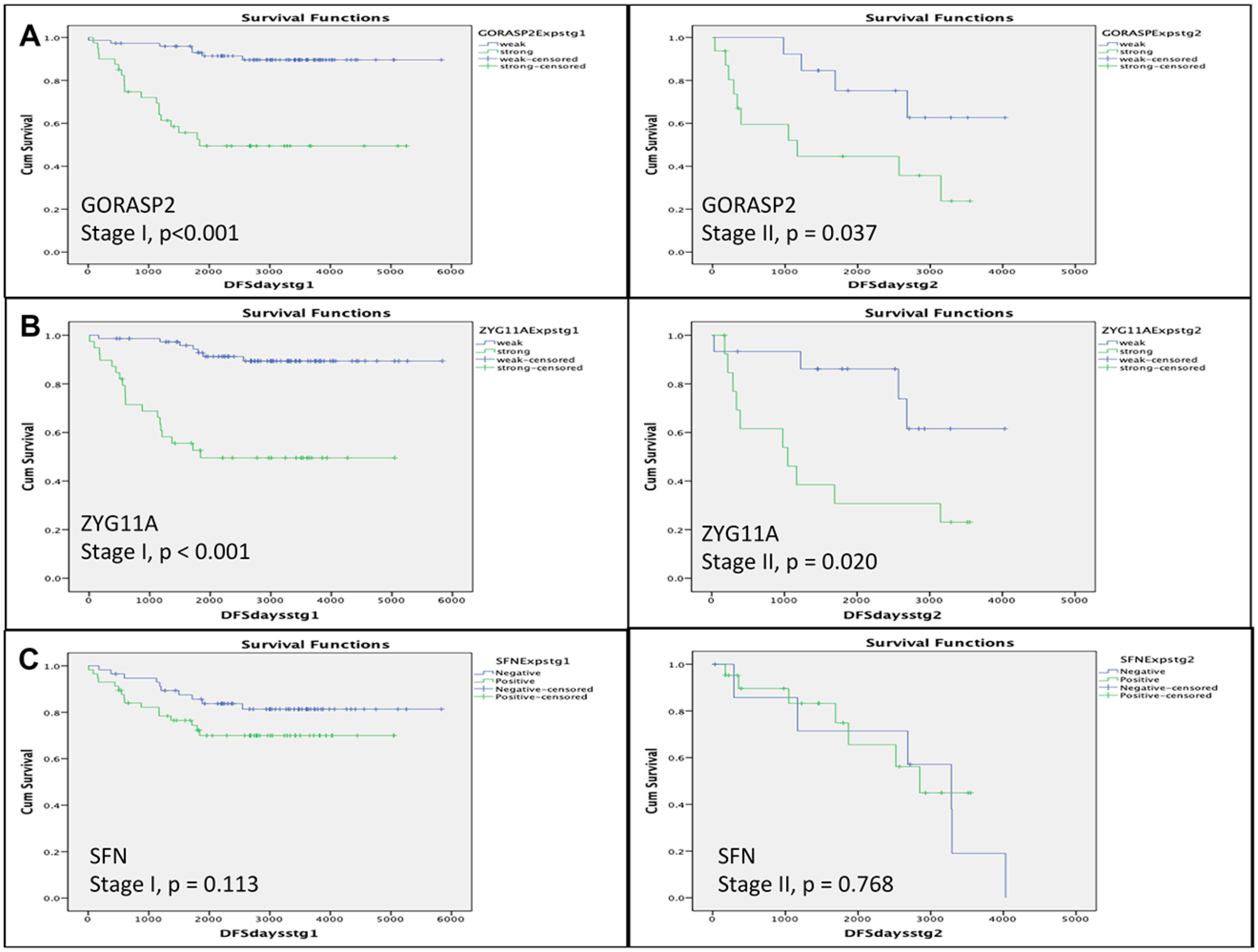

Supplementary Figure 2: KM-curve per stage. Disease-free survival depicted as Kaplan–Meier curves shows significant correlation between gene expression of GORASP2 (**A**), ZYG11A (**B**), but not SFN (**C**) and patients' prognosis in stage I and II, respectively.

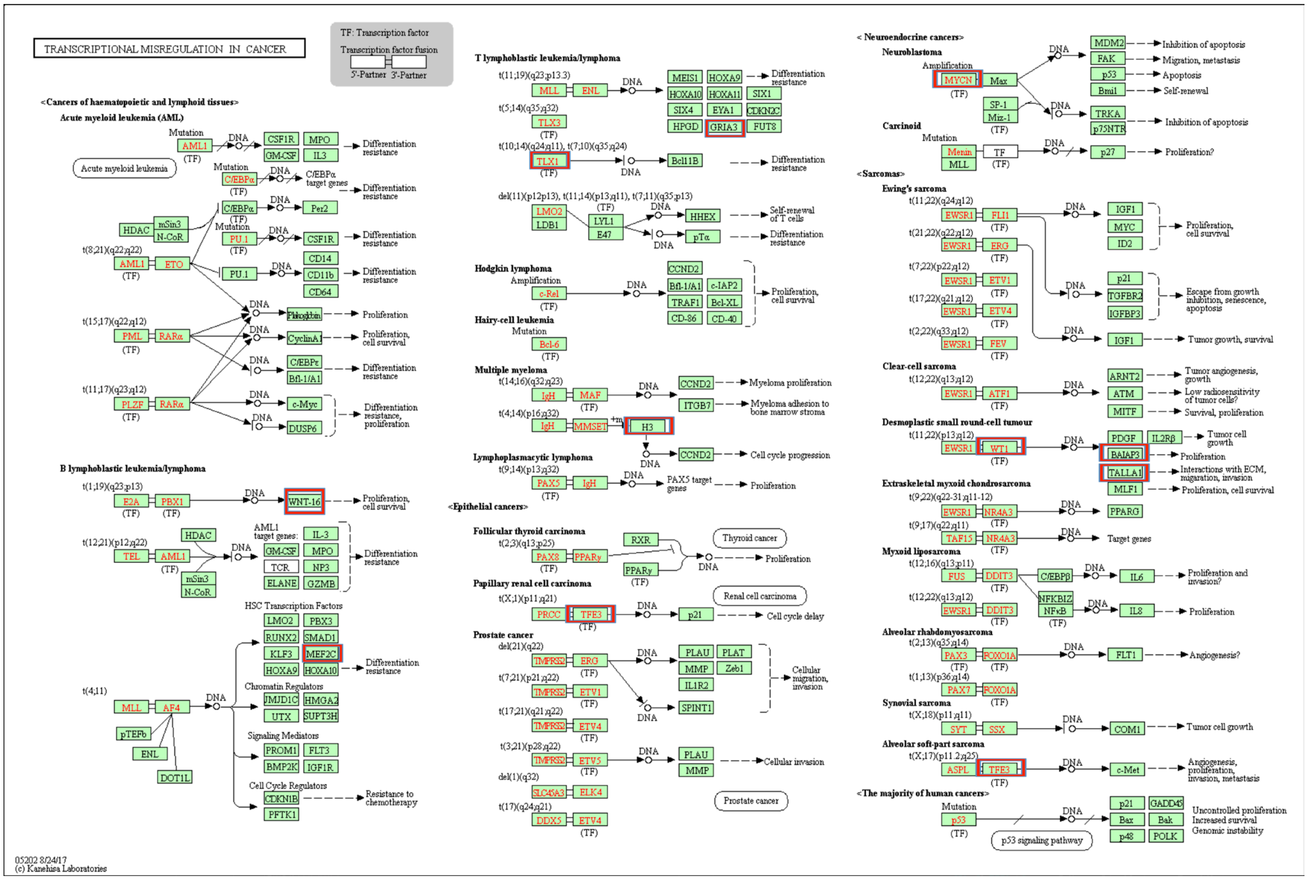

Supplementary Figure 3: Pathway map for transcriptional misregulation in cancer. Red boxes indicates the genes which showed hypermethylation in invasive adenocarcinoma.

Supplementary Table 1: List of hypermethylated genes. See Supplementary_Table_1

Supplementary Table 2: Ingenuity pathway analysis

Ingenuity canonical pathways	*P*-Value
GABA receptor signaling	4.95E-06
Serotonin receptor signaling	9.59E-06
Corticotropin releasing hormone signaling	7.17E-04
Gi signaling	4.41E-03
G-Protein coupled receptor signaling	5.94E-03
Amyotrophic lateral sclerosis signaling	7.34E-03
Neuropathic pain signaling in dorsal horn neurons	9.04E-03
GPCR-mediated integration of enteroendocrine signaling exemplified by L cell	1.03E-02
cAMP-mediated signaling	1.35E-02
RhoGDI signaling	1.55E-02

Supplementary Table 3: KEGG pathway analysis

DAVID	
**KEGG pathways**	***P*-Value**
Neuroactive ligand-receptor interaction	2,8E+10
Nicotine addiction	6,8E+10
Retrograde endocannabinoid signaling	2,9E+11
GABAergic synapse	0.00164
Morphine addiction	0.00253
Mucin type O-glycan biosynthesis	0.00824
Serotonergic synapse	0.02610
Transcriptional misregulation in cancer	0.03198
cAMP signaling pathway	0.03477
Calcium signaling pathway	0.04518

Supplementary Table 4: Hypomethylated gene list

	Beta value											
**Gene name**	**Normal**		**AIS**			**Invasive**			**Mean AIS**	**Mean Invasive**	**Meth Diff**	**CPG sites location**
	1	2	1	2	3	1	2	3				TSS200
BRDT	0.78	0.79	0.66	0.69	0.63	0.45	0.45	0.63	0.66	0.51	0.15	Island
REXO1L2P	0.93	0.97	0.93	0.94	0.95	0.94	0.77	0.51	0.94	0.74	0.20	Island
FLJ39609	0.65	0.73	0.71	0.67	0.69	0.51	0.53	0.67	0.69	0.57	0.12	Island
POM121L12	0.90	0.92	0.92	0.90	0.92	0.90	0.78	0.74	0.91	0.81	0.10	Island
TDRD6	0.84	0.87	0.85	0.85	0.80	0.83	0.65	0.73	0.83	0.74	0.10	Island
GORASP2	0.87	0.83	0.80	0.74	0.61	0.82	0.44	0.44	0.71	0.56	0.15	Shore
HK2	0.76	0.76	0.76	0.68	0.76	0.73	0.47	0.40	0.73	0.54	0.20	Shore
LOC100996579	0.63	0.66	0.36	0.54	0.46	0.18	0.27	0.19	0.46	0.22	0.24	Shore
FBXL7	0.89	0.87	0.74	0.82	0.88	0.85	0.50	0.57	0.81	0.64	0.17	Shore
TSLP	0.81	0.82	0.83	0.75	0.71	0.79	0.48	0.52	0.76	0.60	0.17	Shore
SFT2D3	0.68	0.74	0.73	0.70	0.61	0.76	0.49	0.48	0.68	0.57	0.11	Shore
SRC	0.64	0.69	0.45	0.57	0.57	0.34	0.36	0.57	0.53	0.43	0.11	Shore
ZYG11A	0.72	0.74	0.80	0.71	0.64	0.52	0.51	0.56	0.72	0.53	0.19	Shore
SFN	0.59	0.63	0.36	0.55	0.46	0.25	0.27	0.41	0.46	0.31	0.15	Shore
NLGN2	0.74	0.72	0.73	0.77	0.72	0.78	0.54	0.47	0.74	0.60	0.14	Shore
CNGA4	0.87	0.87	0.88	0.80	0.84	0.83	0.62	0.66	0.84	0.70	0.14	Shore
KCNQ1-AS1	0.71	0.74	0.68	0.71	0.69	0.73	0.52	0.51	0.69	0.59	0.11	Shore
KCNMB3	0.78	0.82	0.81	0.80	0.79	0.80	0.64	0.60	0.80	0.68	0.12	Shore
TTC39A	0.64	0.64	0.48	0.57	0.53	0.30	0.40	0.53	0.53	0.41	0.12	Shore
MIR656	0.77	0.80	0.76	0.77	0.75	0.71	0.62	0.63	0.76	0.65	0.11	Shore
C17orf62	0.77	0.77	0.72	0.69	0.66	0.77	0.57	0.43	0.69	0.59	0.10	Shore
CD1D	0.81	0.84	0.84	0.81	0.76	0.86	0.71	0.52	0.81	0.70	0.11	Shore
TRAF1	0.79	0.79	0.76	0.71	0.77	0.79	0.66	0.29	0.75	0.58	0.16	Shore

Supplementary Table 5: cDNA microarray result

Expression value													
											Mean	Mean	Meth
Gene name	AIS					Invasive					AIS	Invasive	Invasive/
	1	2	3	4	5	1	2	3	4	5			AIS
SFN	1.25	1.41	1.64	1.27	1.99	1.35	4.76	1.11	1.39	1.28	1.51	1.98	1.31
CD1D	0.51	0.54	0.22	0.20	0.01	0.58	0.31	0.68	0.07	0.98	0.27	0.52	1.91
GORASP2	5.51	7.30	6.14	4.59	5.50	4.47	19.65	7.20	8.40	2.62	5.81	8.47	1.46

All 3 genes show more than 1-fold expression value difference between AIS and invasive adenocarcinoma.

Supplementary Table 6: SFN and clinicopathological features

		SFN expression		
**Clinicopathological features**	**Total patients**	**Negative**	**Positive**	***P* value**
**Total Patients**	171	74	97	
**Age (years)**				0.950
<60	42	18	24	
≥60	129	56	73	
**Sex**				0.098
Male	100	38	62	
Female	71	36	35	
**Pathological Stage**				0.062
Stage I	114	57	57	
Stage II	29	8	21	
Stage III	26	9	17	
Stage IV	2	0	2	
**Vascular Invasion**				<0.001*
−	100	56	44	
+	71	18	53	
**Lymphatic Permeation**				0.003*
−	108	56	52	
+	63	18	45	
**Pathological Subtype**				0.001*
AIS	19	14	5	
MIA	29	16	13	
Lepidic	46	17	29	
Acinar	19	9	10	
Papillary	26	13	13	
Solid	32	5	27	

Stage I includes IA and IB, stage II includes IIA and IIB, and stage III includes IIIA and IIIB. Correlation between SFN expression and clinicopathological features was analyzed using chi-squared test.

Supplementary Table 7: GORASP2 and clinicohistopathological features

		GORASP2 expression		
**Clinicopathological features**	**Total patients**	**Weak**	**Strong**	***P* value**
**Total Patients**	171	93	78	
**Age (years)**				0.955
<60	42	23	19	
≥60	129	70	59	
**Sex**				0.009*
Male	100	46	54	
Female	71	47	24	
**Pathological Stage**				<0.001*
Stage I	114	74	40	
Stage II	29	13	16	
Stage III	26	6	20	
Stage IV	2	0	2	
**Vascular Invasion**				<0.001*
−	100	71	29	
+	71	22	49	
**Lymphatic Permeation**				<0.001*
−	108	73	35	
+	63	20	43	
**Pathological Subtype**				<0.001*
AIS	19	19	0	
MIA	29	27	2	
Lepidic	46	28	18	
Acinar	19	8	11	
Papillary	26	4	22	
Solid	32	7	25	

Stage I includes IA and IB, stage II includes IIA and IIB, and stage III includes IIIA and IIIB. Correlation between GORASP2 expression and clinicopathological features was analyzed using chi-squared test.

Supplementary Table 8: ZYG11A and clinicopathological features

		ZYG11A expression		
**Clinicopathological features**	**Total patients**	**Weak**	**Strong**	***P* value**
**Total Patients**	171	98	73	
**Age (years)**				0.293
<60	42	27	15	
≥60	129	71	58	
**Sex**				0.096
Male	100	52	48	
Female	71	46	25	
**Pathological Stage**				0.003*
Stage I	114	75	39	
Stage II	29	15	14	
Stage III	26	8	18	
Stage IV	2	0	2	
**Vascular Invasion**				<0.001*
−	100	69	31	
+	71	29	42	
**Lymphatic Permeation**				<0.001*
−	108	73	35	
+	63	25	38	
**Pathological Subtype**				<0.001*
AIS	19	19	0	
MIA	29	18	11	
Lepidic	46	29	17	
Acinar	19	6	13	
Papillary	26	10	16	
Solid	32	16	16	

Stage I includes IA and IB, stage II includes IIA and IIB, and stage III includes IIIA and IIIB. Correlation between ZYG11A expression and clinicopathological features was analyzed using chi-squared test.
